# Frontier progress of the combination of modern medicine and traditional Chinese medicine in the treatment of hepatocellular carcinoma

**DOI:** 10.1186/s13020-022-00645-0

**Published:** 2022-07-30

**Authors:** Lai Wei, Zeyu Wang, Niancai Jing, Yi Lu, Jili Yang, Hongyu Xiao, Huanyu Guo, Shoukun Sun, Mingjing Li, Daqing Zhao, Xiangyan Li, Wenxiu Qi, Yue Zhang

**Affiliations:** 1 College of Traditional Chinese Medicine, Changchun University of Chinese Medicine, Changchun, 130117 Jilin China; 2 Department of Scientific Research, Changchun University of Chinese Medicine, Changchun, 130117 Jilin China; 3 Department of Integrated Chinese and Western Medicine, Jilin Cancer Hospital, Changchun, 130000 Jilin China; 4 Northeast Asia Research Institute of Traditional Chinese Medicine, Key Laboratory of Active Substances and Biological Mechanisms of Ginseng Efficacy, Ministry of Education, Jilin Provincial Key Laboratory of Bio-Macromolecules of Chinese Medicine, Changchun University of Chinese Medicine, Changchun, 130117 Jilin China

**Keywords:** Traditional Chinese medicine, Hepatocellular carcinoma, Combination therapy, Adjuvant therapy, Chemotherapy, Molecular targeted therapy, Multidrug resistance, Apoptosis, Antitumor, Overall survival

## Abstract

Hepatocellular carcinoma (HCC, accounting for 90% of primary liver cancer) was the sixth most common cancer in the world and the third leading cause of cancer death in 2020. The number of new HCC patients in China accounted for nearly half of that in the world. HCC was of occult and complex onset, with poor prognosis. Clinically, at least 15% of patients with HCC had strong side effects of interventional therapy (IT) and have poor sensitivity to chemotherapy and targeted therapy. Traditional Chinese medicine (TCM), as a multi-target adjuvant therapy, had been shown to play an active anti-tumor role in many previous studies. This review systematically summarized the role of TCM combined with clinically commonly used drugs for the treatment of HCC (including mitomycin C, cyclophosphamide, doxorubicin, 5-fluorouracil, sorafenib, etc.) in the past basic research, and summarized the efficacy of TCM combined with surgery, IT and conventional therapy (CT) in clinical research. It was found that TCM, as an adjuvant treatment, played many roles in the treatment of HCC, including enhancing the tumor inhibition, reducing toxic and side effects, improving chemosensitivity and prolonging survival time of patients. This review summarized the advantages of integrated traditional Chinese and modern medicine in the treatment of HCC and provides a theoretical basis for clinical research.

**Supplementary Information:**

The online version contains supplementary material available at 10.1186/s13020-022-00645-0.

## Introduction

**Fig. 1:**
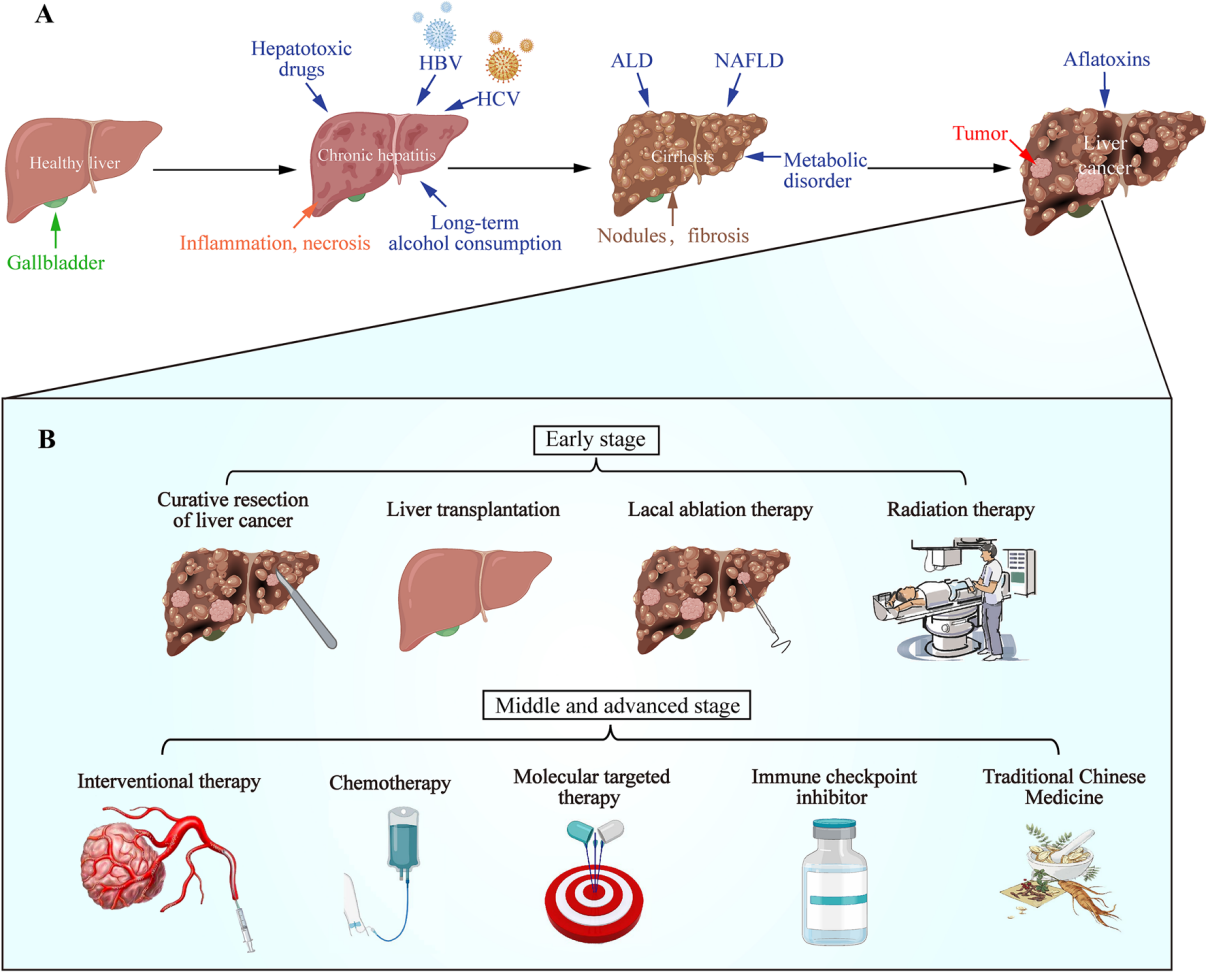
Trilogy of occurrence and development for HCC and current treatment **A** The cause of HCC is not very clear, but the main risk factor is chronic hepatitis caused by hepatitis B virus or hepatitis C virus. At the same time, due to heavy drinking and drug toxicity, could cause liver cirrhosis, ALD, NAFLD, metabolic abnormalities (such as type 2 diabetes), liver fibrosis and aflatoxin contaminated food, all of the above liver diseases may lead to HCC eventually. **B** Patients with a single or within 3 liver tumors, the tumor diameters less than 3 cm are classified as early cancer (stage I), also known as small HCC, and could be get better prognosis from liver resection, liver transplantation, local ablation, and radiation therapy. Patients with greater tumor, limited to the liver and Child Pugh A/B are considered to have intermediate stage cancer (stage II), which could be benefit from interventional therapy (IT) and chemotherapy. HCC patients with vascular invasion and/or extrahepatic cancer symptoms are considered to have advanced cancer (stage III) which profits by molecular targeted therapy (sorafenib), immune checkpoint inhibitors and TCM treatment based on syndrome differentiation. Patients with end-stage (stage IV) HCC usually show Child Pugh C/D or PS 3–4, so they take palliative treatment

Hepatocellular carcinoma (HCC) was the sixth most common cancer in the world in 2020 and the third leading cause of cancer death, after lung cancer and colorectal cancer, accounting for 8.3% of all cancer deaths [1]. China was the highest incidence rate of HCC. The new cases of HCC in China accounted for 45.3% of the world's total in 2020 [1]. The cause of HCC remains unclear, but the main risk factor was chronic hepatitis caused by hepatitis B virus or hepatitis C virus [2, 3]. In addition, heavy drinking, aflatoxin contaminated food, and drug toxicity could cause liver cirrhosis, alcoholic liver disease (ALD), non-alcoholic fatty liver disease (NAFLD), metabolic abnormalities (such as type 2 diabetes), and liver fibrosis, all of which may lead to HCC eventually [4–6] (Fig. 1A). Primary liver cancer could be divided into HCC, intrahepatic cholangiocarcinoma (ICC), and HCC-ICC mixed type according to pathological and histological sources [7]. HCC accounted for 85%-90% of primary HCC [4].

The diagnosis of HCC was based on the results of biopsy or imaging combined with molecular marker analysis [8]. According to the guidelines for diagnosis and treatment of primary HCC, the stage of HCC was mainly determined by the number and size of liver tumors, vascular invasion, extrahepatic metastasis, Child Pugh grade and performance status (PS) [9]. Patients with a single or less than 3 liver tumors and a tumor diameter less than 3 cm were classified as early cancer (stage I), also known as small HCC, and get good prognosis after liver resection, liver transplantation, local ablation, and radiation therapy [10–13]. Patients with greater tumor, limited to the liver and Child Pugh A/B are considered to have intermediate stage cancer (stage II), and benefit from IT and chemotherapy [14, 15]. HCC patients with vascular invasion and/or extrahepatic cancer were considered to have advanced cancer (stage III), and benefit from molecular targeted therapy, immune checkpoint inhibitors and TCM treatment based on syndrome differentiation [16–18]. Patients with end-stage (stage IV) HCC usually showed Child Pugh C/D or PS 3–4, and thus receive palliative treatment [19] (Fig. 1B). However, clinically, due to the lack of clear early markers of HCC, most patients were not suitable for surgery and liver transplantation in the middle and late stage of HCC. IT (including radiofrequency ablation, RFA and transcatheter arterial chemoembolization, TACE) was easy to cause local recurrence and post embolism syndrome (PES) [20, 21]. Immunotherapy and molecularly targeted medications were vulnerable to drug resistance, adverse effects from therapy, and an unsatisfactory objective remission rate of 15% to 20% [22–24]. Given the above-mentioned shortcomings of current clinical treatment approaches, the addition of TCM enhanced the comprehensive anti-tumor effect, and therefore emerges as a focus of HCC treatment [25–27]. Multiple experiments have investigated the mechanisms of TCM in inhibiting HCC. Alperine inhibited Akt-mediated apoptosis, G2/M cell cycle arrest and the proliferation of hepatoma cells [28]. Lappaconitine sulfate induced apoptosis by mediating the mitochondrial apoptosis pathway [29]. Bufalin regulated tumor immune microenvironments by Nuclear Factor kappa B (NF-κB) and activates anti-tumor T cell immune response [30]. Breviscapine alleviated nonalcoholic steatohepatitis and liver fibrosis by inhibiting TGF-β-activated kinase 1 and toll-like receptor 4 (TLR4) / NF-κB signaling pathway to protect the liver and prevent the further development of liver disease to HCC [31, 32]. Astragaloside IV inhibited macrophage M2 polarization, invasion and proliferation of hepatocellular cells by regulating TLR4 / NF-κB / signal transducer and activator of transcription 3 (STAT3) signaling pathway [33]. In addition, Astragaloside IV and Curcumin down-regulated the expression of fibroblast growth factor 2, matrix metalloproteinase 2 (MMP2), vascular endothelial growth factor (VEGF), hepatocyte growth factor, and synergistically inhibited nude-mouse model of HCC tumor growth and angiogenesis [34]. The above research results demonstrate the huge potential of TCM to combat HCC.

Furthermore, TCM, as an adjuvant therapy, could reduce adverse reactions, increase the curative effect, and prolong the survival time of patients [35]. Combination therapy had become a popular strategy for the treatment of HCC. So, this review was divided into two parts. First, the antitumor effect of TCM combined with clinical medication since the establishment of the literature database was summarized. The synergistic effect of TCM combined with surgery, IT, chemotherapy, and targeted drugs in clinical studies were sorted in the second part. It is found that TCM could be used as an adjuvant therapy to intervene in HCC, so as to lay a foundation for the clinical treatment of HCC and the in-depth research of integrated TCM and modern medicine in the treatment of HCC in the future.

## Methods

This study included basic and clinical studies of TCM combined with arbitrary clinical therapy or drug for HCC published in English as of October 2021 since the establishment of PubMed and Web of Science. We searched 4 sets of keywords in the title and abstract, such as (1) “traditional Chinese medicine” OR “active ingredient” OR “decoction” OR “capsule” OR “injection” OR “formula” OR “granule”; (2) “combine” OR “synergistic” OR “adjuvant treatment”; (3) “prevent” OR “relieve” OR “improve” OR “resistance”; (4) “hepatocellular carcinoma” OR “liver cancer”. Used "AND" combination 4 sets of retrieval results: (1) AND (2) AND (3) AND (4). Search again for the list of references for articles that fulfilled the inclusion criteria after importing the retrieved articles into Endnote X9 to weed out duplicates and filtered them through inclusion and exclusion criteria.

### Inclusion criteria

The inclusion criteria for these retrieved papers were English publications that have been published or have study findings. The study methods were clinical and basic research. TCM interventions included all extracts and preparations (monomer, active ingredient, compound, capsule, granule, and injection). Combined interventions included commonly used clinical modern medical treatment (chemotherapy, interventional therapy, molecular targeted therapy, and immunotherapy). Clinical research included clinical observation, clinical control study, prospective, and retrospective study. Regardless of age or gender, each subject received a primary liver cancer diagnosis.

### Exclusion criteria

The language type of the article was not English. Articles on intervention of HCC only with or without TCM were excluded. The disease types of intervention were liver metastasis or other types of tumors. Article types for literature review, meta-analysis and case report were also excluded.

### Data extraction

Two researchers (LW and ZYW) independently reviewed the retrieved articles, further assessed and extracted data from articles that met the inclusion criteria. Any discrepancies or doubts regarding the data were cleared up through conversation or contact with a third researcher (WXQ).

Basic researches gathered the following information: article title, the type of TCM intervention and detailed information (name, composition or compound ingredients), combination drugs, cellular/animal model, pathway/target and effect.

**Fig. 2:**
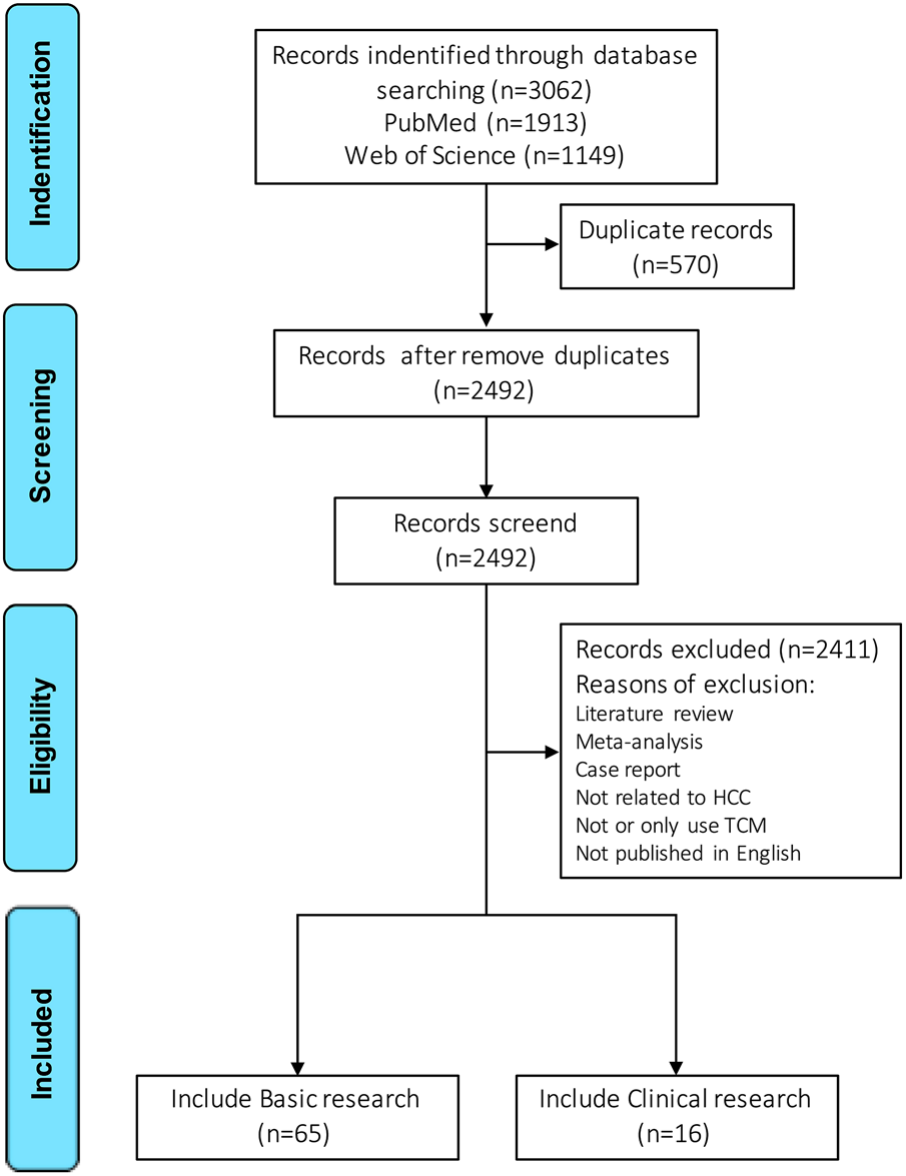
The flow chart of the articles collection This study included basic and clinical studies of TCM combined with arbitrary clinical therapy or drug for HCC published in English as of October 2021 since the establishment of PubMed and Web of Science. The keywords such as "Traditional Chinese medicine", "hepatocellular carcinoma", "combination" and "resistance" were searched to screen articles with inclusion and exclusion criteria. Finally, 65 basic research articles and 16 clinical articles were collected

Clinical researches gathered the following information: article title, the type and detailed information of TCM intervention (name, composition or compound ingredients, dosage), combination drugs/therapy, intervention time (if any), sample size, sample source, primary outcome, secondary outcome and effect. The flow chart of the articles collection is shown in Fig. 2.

### Current situation of therapeutic drugs for HCC

In the basic study of clinical drugs for the treatment of HCC, it was found that mitomycin C (MMC), doxorubicin (DOX), cyclophosphamide (CTX) and 5-fluorouracil (5-FU) affect the development of tumor by interfering with DNA synthesis [36–39]. However, with the use of drugs, it was found that DOX and 5-FU were prone to severe cardiotoxicity, and the cytotoxicity caused by CTX usually leads to bone marrow suppression and immunosuppression [37, 40–42]. Platinum chemotherapeutic drugs interfered with DNA repair mechanism, led to DNA damage, and then induced cancer cell apoptosis [43, 44]. Sorafenib was an oral multi-kinase inhibitor, targeting VEGF receptor, RAF and PDGF receptor, which had anti-angiogenesis and direct anti-tumor effects [45]. Platinum chemotherapy drugs and sorafenib were prone to drug resistance, and there were serious adverse reactions after long-term use [46–48]. ICIs such as anti-programmed cell death protein 1 (PD1) antibody and its ligand (PD-L1) are a new method for clinical treatment of HCC [49]. However, immune related adverse events (irAEs) had become a difficult problem in clinical practice, the main clinical manifestations are skin toxicity, liver toxicity and gastrointestinal toxicity [50]. In the face of the above-mentioned clinical side effects of chemotherapeutic drugs commonly used in HCC and the characteristics of drug resistance, the treatment of HCC is very passive. Fortunately, TCM played a corresponding role in the gastrointestinal reactions and toxic side effects caused by chemotherapy. Therefore, it seems that the intervention of TCM could achieve a more optimized treatment outcome.

### Combination of TCM and MMC

Hau DouMong et al. found that Shih Chuan-Ta-Pu-Tang (SCTPT) or Shi-Hung-One (SHO) combined with MMC could prolong the survival time of tumor-bearing mice [51]. The combination of polyporus umbellatus extract and MMC improved the life span of S180-tumor bearing mice, changed the tumor micro-environment, increased lymphocyte infiltration, induced cancer cell atrophy and fibrosis by inhibiting the synthesis of DNA, RNA and protein [52]. Xuefu Zhuyu tang combined with MMC could specifically inhibit the survival rate of tumor cells and prolong the average survival time of tumor-bearing mice [53]. Early studies on MMC found that combining it with herbal extract or a Chinese medicine ingredient improved its therapeutic effects and lengthened the lives of tumor-bearing mice by boosting their immune systems. (Additional file 1: Table S1).

### Combination of TCM and CTX

The research found that neutral polysaccharide from Panax notoginseng (NPPN) alleviated the bone marrow suppression caused by CTX treatment, inhibited the growth of tumor in mice bearing H22 tumor, improved the antitumor effect of CTX and alleviated adverse reaction [54]. TCM classic prescription Shenling Baizhu Powder combined with CTX could reduce the expression of NF-κB, B-cell lymphoma-2 (Bcl-2)/B-cell lymphoma-extra large (Bcl-xl), survivin and X-linked inhibitor of apoptosis (XIAP) in tumor tissues in mice, which promoted the activation of caspase-3/9, so as to inhibit tumor growth [55]. Shengbai Decoction reduced the content of inflammatory factors in the serum of mice after CTX and was found to promote the apoptosis of HCC cells through p53 pathway, and reduce side effects and prolong survival [56]. It was found that He-Wei Granule combined with CTX reduced the hepatotoxicity and bone marrow inhibition caused by CTX, increased cleaved caspase-3 to promote apoptosis and inhibit autophagy [57]. The studies above showed that TCM could enhance CTX, promote the apoptosis of hepatoma cells. The combination improved the antitumor effect, reduced the toxic and side effects of CTX, and finally improved the survival time of tumor-bearing mice (Additional file 1: Table S1).

### Combination of TCM and DOX

Both icaritin and ginger extract promoted DOX and induced apoptosis of hepatoma cells. And ginger extract reduced malondialdehyde and tumor necrosis factor (TNF)-α to improve the cardiotoxicity caused by DOX [58, 59]. Dihydroartemisinin, Y_6_ (epigallocatechin gallate derivative) and astragalus polysaccharides overcame the drug resistance of DOX and restored its sensitivity in the treatment of HCC, while astragalus polysaccharides could regulate the expression level of serum cytokines in mice bearing H22 tumor and inhibit tumor progression [60–62]. Rosmarinic acid, cinobufacini and quercetin separately synergized DOX to induce the apoptosis of hepatoma cells and boost the therapeutic effect of DOX [63–65]. Dahuang Zhechong Pill (DHZCP) is a classic TCM prescription, which mediated the multidrug resistance (MDR) of HCC and reduced the outflow of drugs by improving the activity level of adenosine triphosphate (ATP) in SMMC-7721 cells, and thereby enhanced the effect of DOX [66]. Meanwhile, DHZCP inhibited angiogenesis, promoted apoptosis and reversed resistance on DOX-resistant SMMC-7721 xenografts in mice [67]. The active ingredient of Tanshinone IIA could prolong the survival time of tumor-bearing mice, reduce the expression of cytochrome P450, cytochrome P450 Family 3 Subfamily A Member 4 (CYP3A4) and pregnane X receptor (PXR), and reduce the toxicity of DOX and the level of serum aspartate transaminase (AST) [68]. Tanshinone IIA could also improve the tumor micro-environment by reducing tumor hypoxia and regulating the expression of α-Smooth muscle actin(α-SMA) and collagen IV to remodel the tumor vasculature and inhibit angiogenesis after combined treatment [69]. These studies showed that TCM could alleviate the cardiotoxicity of DOX. The combination further inhibited tumor formation in tumor-bearing mice by promoting apoptosis and inhibiting the proliferation of HCC cells and improved the sensitivity of DOX in the treatment of HCC (Additional file 1: Table S1).

### Combination of TCM and DDP

When falcarindiol or astragaloside IV was combined with DDP separately, the sensitivity of tumor-bearing mice to DDP and the antitumor effect of DDP by inducing apoptosis of HCC cells could be enhanced [70, 71]. It was found that matrine could reduce the toxicity of DDP, induce apoptosis of hepatoma cells in tumor-bearing mice through reducing the expression of survivin [72]. Bushen Huayu Jiedu recipe combined with DDP was administered to H22 tumor-bearing mice, the high-dose group showed obvious repression rate on the mean weight of tumor, which was better than that of DDP alone (77.69% vs 68.46%) [73]. In clinic, two or more chemotherapeutic drugs are often used in refractory HCC to improve sensitivity, which, however, also brings increased toxicity [39]. Researchers found that wheat germ extract could inhibit the proliferation of a variety of HCC cells, induce the expression of poly ADP-ribose polymerase (PARP) in Hep3B cells, cause apoptosis, and enhance the inhibitory effect of DDP and 5-FU on HCC cells [74]. Solanum nigrum, a TCM with antipyretic and analgesic effects, is a commonly used antitumor drug [75]. Wang Chienkai et al. found that aqueous extract of *Solanum nigrum* (AE-SN) could promote the apoptosis of Hep3B and HepJ5 by inducing cleaved caspase-7, activating intracellular autophagy, and enhancing the cytotoxicity of DDP and DOX to hepatoma cell lines [76]. L-OHP, as one of the third generation of platinum drugs, do not produce cross resistance to DDP [77]. Researchers found that the purified polysaccharide extracted and isolated from the fruit bodies of *L. edodes* combined with L-OHP induced apoptosis and inhibited the activity of cancer cells, inhibited the angiogenesis and reduced the adverse reactions such as diarrhea and liver injury in tumor-bearing mice [78]. The hepatoma cells treated with L-OHP alone did not significantly induce apoptosis, accompanied with high expression of Yes associated protein (YAP) [79]. However, its combination with Huaier induced the apoptosis of hepatoma cells [79]. It has been found that demethylcantharidin precluded the repair of cisplatin-induced DNA cross-links by inhibiting protein phosphatase 2A (PP2A) to avoid the resistance to cisplatin in mice with hepatoma [80]. The administration sequence of demethylcantharidin in advance of cisplatin had a lower combination index than that of cisplatin in advance of demethylcantharidin in SK-Hep-1 cells (0.72 ± 0.07 vs 0.99 ± 0.08), which produced a significant synergistic effect in vitro [80]. Therefore, experimental studies showed that TCM promoted the apoptosis of hepatoma cells treated with platinum drugs, overcame the MDR, and improved the nephrotoxicity and gastrointestinal side effects of platinum drugs on tumor-bearing mice (Additional file 1: Table S1).

### Combination of TCM and 5-FU

ZYGII (a kind of triterpenoid saponins isolated from *Sanguisorba officinalis L*), pleurospermum lindleyanum extract and furanodiene (a sesquiter mec pene component in zedoary turmeric rhizome) could regulate the cell cycle process, induce apoptosis and promote the anticancer effect of 5-FU in hepatoma cells [81–84]. Furadiene resisted angiogenesis and induced the production of reactive oxygen species (ROS) [83, 84]. Ciji-Hua’ai-Baosheng II Formula could improve the anorexia and gastrointestinal injury caused by chemotherapy in tumor-bearing mice by affecting the appetite regulatory factors in the hypothalamic central nervous system and feeding area, and enhance the antitumor effect of 5-FU [85]. Curcumin is an anticancer component of turmeric. It could inhibit the proliferation of HCC cells and improve the sensitivity of 5-FU chemotherapy by increasing the intestinal microbiota of mice when combined with 5-FU [86]. The total water-soluble flavonoids isolated from *Isodon lophanthoides var. gerardianus (Benth.) H. Hara* combined with 5-FU to inhibit tumor activity by promoting apoptosis and improving ROS level [87]. Li Fengli et al. found that H1 (a derivative of tetrandrine) could increase the cytotoxicity of 5-FU in BEL7402/5-FU cells by blocking STAT3/myeloid cell leukemia-1 (Mcl-1) pathway and inducing p53 up-regulated modulator of apoptosis (PUMA), reverse the resistance to 5-FU and further promote apoptosis of drug-resistant cells [88]. Huaier inhibited the proliferation of HuH28 by down regulating STAT3 as well as its downstream genes, and had stronger effects in cell cycle arrest, induced-apoptosis and anti-metastasis when combined with 5-FU [89]. The combination of puerarin, or luteolin, or resveratrol with 5-FU enhanced the apoptosis of hepatoma cells, which might be related to the decreased activity of dihydropyrimidine dehydrogenase (DPD) in 5-FU metabolism [90–93]. Therefore, TCM combined with 5-FU could cause cell cycle arrest, promote apoptosis, enhance oxidative stress, and prevent the continuous proliferation of HCC cells. Additionally, TCM reduced the adverse reactions such as anorexia caused by 5-FU (Additional file 1: Table S1).

### Combination of TCM and sorafenib

It was found that both Bushen Jianpi Formula and cucurbitacin B induced apoptosis of HCC cells by regulating the levels of caspase-3 and caspase-9, and inhibited the proliferation of tumor cells in vivo in combination with sorafenib [94, 95]. Bufalin or Celastrol (major active ingredient of Tripterygium wilfordii) combined with sorafenib reduced the secretion of VEGF, inhibited tumor angiogenesis in mice bearing tumor and enhanced the activity of sorafenib [96–99]. Curcumin and Oridonin (the core bioactive component of rabdosia rubescens) could block the epithelial–mesenchymal transition (EMT) and overcome the limitations of sorafenib [100, 101]. Magnolol (a bioactive compound extracted from the bark of the Magnolia officinalis) and LicA (Licochalcone A, the main component of Licorice) enhanced the inhibition of the invasion of HCC cells by sorafenib in vivo and in vitro [102, 103]. Besides, LicA was found to reduce the incidence of lung metastasis in tumor-bearing mice [103]. Compound kushen injection (CKI, also known as Yan shu injection), an anticancer Chinese patent medicine, activated immunosuppression, reshapes the immune microenvironment of HCC and enhanced the antitumor activity of sorafenib [104]. Berbamine (a natural bisbenzylisoquinoline alkaloid isolated from Berberis amurensis) or ouabain cooperated with sorafenib to reduce the expression of epidermal growth factor receptor (EGFR) and insulin-like growth factor receptor (IGF1R) and to inhibit the growth of hepatoma cells by inhibiting sarcoma gene (Src). Berbamine also improved the sensitivity of sorafenib resistant to HepG2-SR cells [105]. Hydroxygenkwanin (HGK), a natural herbal extract from Daphne genkwa, inhibited the activity of class I histone deacetylase (HDAC), which could improve the therapeutic effect after combined with anti-cancer drugs such as sorafenib [106]. Artesunate (artemisinin derivative extracted from Artemisia annua) significantly enhanced the anticancer effect of sorafenib on HCC cell lines in vitro and mice bearing Huh7 tumor, which was attributed to the synergistic effect of lysosomal activation induced by oxidation, induced ferroptosis and improved the sensitivity of sorafenib to HCC cells [107]. In conclusion, TCM combined with sorafenib inhibited the proliferation and invasion of HCC cells through inducing apoptosis and blocking the EMT, and improved the sensitivity of sorafenib to HCC cells (Additional file 1: Table S1).

In addition, the combination of TCM with ICIs, other chemotherapeutic drugs and molecular targeted drugs improved the antitumor effect of the drugs [108–113] (Additional file 1: Table S1).

### TCM overcomes MDR

With the increase of treatment cycle, the drug resistance of HCC to cytotoxicity affects the effect of chemotherapy, and the main mechanism is MDR [114]. As an adjuvant drug after chemotherapy, TCM could prolong the survival time of cancer patients, which might be related to its obstruction of MDR related pathways [115]. Cantharidin inhibited the MDR1 gene, blocked the P-gp pathway, and reversed MDR [116]. Gambogenic acid, an extract of Gamboge, could mediate NF-κB and MAPK pathway through P-gp, promote cell apoptosis, block MDR and improve the sensitivity of hepatoma cells resistant to DOX and paclitaxel [117]. Another natural compound Asiatic acid Naringenin (AANG) targeted the tumor micro-environment, inhibited transforming growth factor-β1 (TGF-β1)/Smad signaling, and reduced the MDR caused by P-gp [118]. Apigenin, a natural flavonoid, reduced inflammation and oxidation, down-regulated the expression of HIF-1α through the inhibition of protein kinases B (PKB, also known as Akt)/p-Akt pathway and Hsp90, and overcame the drug resistance of paclitaxel induced by hypoxia [119]. Chinese herbal extract platycodin D reversed the resistance of HCC cells to chemotherapeutic drugs through ERK1/2 pathway [120]. In conclusion, the extracts from the above herbs could block the occurrence of MDR, HIF-1, P-gp and inflammation related pathway (Additional file 1: Table S2).

### Clinical study of TCM combined with modern medicine in the treatment of HCC

Hepatectomy and liver transplantation are good choices in the early stage of HCC, but they are only applicable for a few patients and with a high risk of postoperative recurrence [121]. IT is beneficial for patients with middle-term HCC, but prone to local complications and post embolization syndrome (PES) [21]. Advanced HCC is usually treated with routine treatment or symptomatic treatment in order to prolong the survival time of patients [122]. In light of the current situation of clinical treatment of HCC, TCM adjuvant treatment seems to show better outcome indicators.

### TCM combined with Surgery for HCC

In a RCT after an operation of 364 patients with small HCC from 5 centers, cinobufagin and Jiedu Granule (THM group) showed great advantages in prolonging the recurrence time and improving the survival rate over TACE. The median recurrence-free survival (RFS) was 46.9 vs 34.4 months (*P* = 0.048), Cox proportional hazard regression analysis showed that THM (hazard ratio [HR] = 0.57,95% confidence interval [CI], 0.37–0.86) was an independent protection factor affecting overall survival (OS). The rate of 5-years OS was 71.11% vs 63.04%, respectively [123]. In another phase IV study of 1044 patients after HCC surgery from 39 centers, the mean RFS was 75.5 vs 68.5 weeks (HR 0.67, 95% CI, 0.55–0.81), and the 2-years OS rate was 95.19% vs 91.46% (HR = 0.553, 95% CI, 0.33–0.92) in the Huaier group treated with Huaier Granule and in the control group. In addition, Huaier Granule treatment significantly reduced the recurrence and metastasis rate of HCC in patients without HBV infection [124]. In a retrospective study of 137 patients after HCC surgery, it was found that the recurrence rate of HCC treated with Erzhu Qinggan Jiedu recipe (ESQJR) combined with modern medicine was reduced by 22.6% (57.4% vs 34.8%, *P* = 0.008), and the average OS was significantly longer than that of modern medicine alone (139.9 vs 97.2 months, *P* = 0.043) [125]. A clinical study of 120 cases found that Jianpi Huayu therapy prolonged the median OS (52.6 vs 49.8 months, *P* = 0.048) and median disease-free survival (28.7 vs 22.6 months, *P* = 0.045) of postoperative HCC patients compared with the control group, and the recurrence rate was significantly lower than that of the control group (80% vs 93.9%) [126]. These studies proved the effectiveness of TCM on postoperative intervention of HCC, delayed the progress of the disease, and significant improvement of the survival time of postoperative patients (Additional file 1: Table S3).

### TCM combined with IT for HCC

The combination of Jianpi Ligan Decoction (JLD) and TACE (n = 103) or RFA (n = 95) reduced the adverse effects and improved the success rate of treatment, and the 3-year OS rate was improved compared with only TACE (37.74% vs 26.00%, HR = 0.62, 95% CI, 0.38–0.99) or only RFA (38.30% vs 27.08%, HR = 1.78, 95% CI, 1.08–2.93) [127, 128]. In the cohort study of 340 HCC patients, compared with the thermal ablation (RFA or microwave ablation [MWA]) group, the Huaier Granule combined with thermal ablation showed an increase in both the median OS (35 vs 31 months, HR = 0.76, 95%CI, 0.54–1.07) and median progression-free survival (PFS) (24 vs 12.5 months, HR = 0.67, 95%CI, 0.48–0.94), and a significant decrease in the rate of extrahepatic metastasis, which could alleviate the side effects of patients to some extent [129]. The study found that the rate of 3-year OS of the Chaihu-Huaji Decoction combined with the TACE group was significantly higher than that of the control group (26.47% vs 13.06%, HR = 1.61, 95%CI, 1.02–2.53), without hepatorenal toxicity [130]. After cantharidin capsule was administered with TACE, the overall effective rate increased (40.6% vs 36.8%, *P* < 0.001), with an improved Karnofsky performance status (KPS), a reduced alpha fetoprotein (AFP) value, a reduced gastrointestinal reaction and an improved quality of life of patients compared with the control group [131]. After a long-term follow-up of 399 patients, Compound Ruanjian Hugan tablet (RJH) was found to play a positive role in the treatment of small HCC. The median OS of RJH combined with the IT group was better than the IT group and simple operation group (151.20, 43.8 vs 20.77 months). The combined group compared with the IT group, the 10-year OS rate was 57.10% and 33.34% [132]. In a prospective study of Ginsenoside Rg3 was combined with TACE in 228 HCC patients, compared with the control group, the median OS was 13.2 vs 10.1 months (HR = 0.63, 95% CI, 0.46, 0.85) and a higher disease control rate of 69.7% vs 51.3%(*P* = 0.012) was achieved [133]. In a RCT of 98 HCC cases, Jinlong Capsule combined with TACE had a much higher overall remission rate (60.38% vs 40%, *P* > 0.05), and a decreased KPS and levels of serum osteopontin (OPN) compared with TACE alone. The combination also inhibited the progress of the disease and improved the prognosis [134]. In a study with a follow-up for 86 months with 266 HCC patients, Jiedu Granule (JD) combined with TACE prolonged time to progress (TTP) (8.67 vs 5.37 vs 4.57 months) and the median OS (21.43 vs 23.23 vs 13.97 months) compared with TACE alone and TACE plus sorafenib [135] (Additional file 1: Table S4). It followed that the combination of TCM prolonged the PFS and OS, increased the effective rate of IT and improved the adverse reactions of patients with HCC.

### TCM combined with CT for HCC

Phase II study found that YIV-906 combined with capecitabine (an oral 5-FU prodrug [136]) had the median OS of 6 months in patients with 39 HCC and showed better clinical effects on patients with low initial AFP value, those uninfected by HBV and those previously untreated [137]. A retrospective cohort study involving 328 cases found that TCM combined with conventional therapies (include TACE, ablation, target therapy, or chemotherapy) was superior to TCM alone or CT alone, with the median OS of 11 vs 8.6 vs 9.4 months(*P* < 0.01). Multivariate analysis showed that integrative therapy belonged to the protective factor and had a lower risk of death in contrast to the CT group (HR = 0.59, 95% CI 0.42–0.84) or CHM group (HR = 0.59; 95% CI, 0.46–0.77). Under integrative therapy, Child Pugh class A and better PS could benefit from OS, which suggested that patients with HCC benefited from early TCM medicine intervention [138]. Liao Yueh-Hsiang et al. conducted a retrospective study on 127,237 HCC cases in Taiwan Health Insurance Research Database and found that Jiawei Xiaoyao san (HR = 0.89, 95% CI, 0.81–0.96) and Chaihu Shugan Decoction (HR = 0.86, 95% CI = 0.78–0.95) were effective TCM preparations in improving the survival rate of patients with HCC, and the mean follow-up period was 5.67 vs 5.49 years for TCM and non-TCM users, respectively. Compared with non-TCM users, TCM reduced the risk of death (HR = 0.65, 95% CI, 0.64–0.66)*.* Meanwhile, TCMs were found to have significant protective effects in different subgroups of patients with chronic liver diseases [139] (Additional file 1: Table S5).

**Fig. 3:**
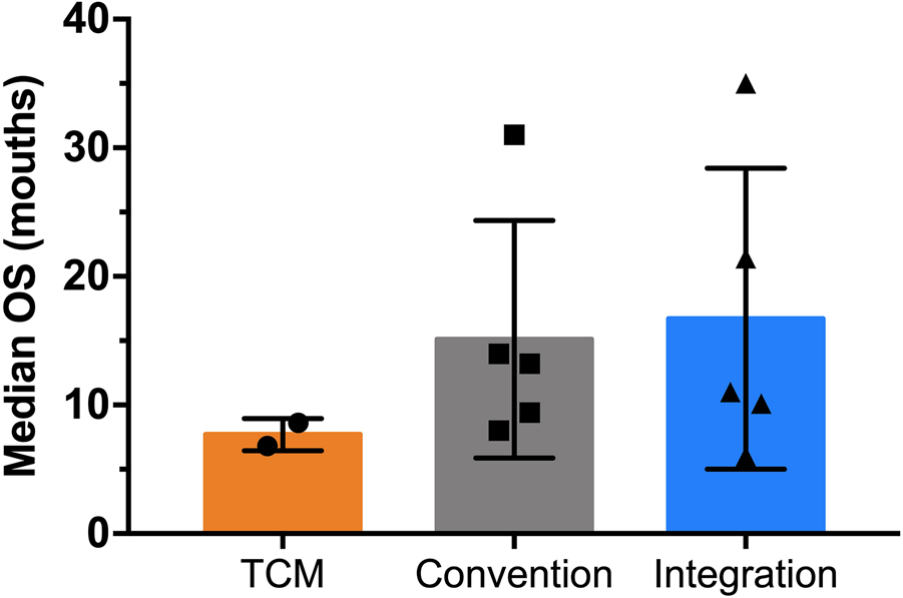
The median OS data for advanced HCC Gathered and compiled the median OS data from the clinical data of integrated traditional Chinese and modern medicine in the treatment of advanced HCC. The median OS of the Integration group was higher than that of the Convention group and the TCM group, which was 16.71 vs 15.11 vs 7.72 months (*P* > 0.05)

We gathered and compiled the median OS data from the clinical data of integrated traditional Chinese and modern medicine in the treatment of advanced HCC [27, 129, 133, 135, 137, 138]. Taking the use of TCM as an exposure factor, they were divided into the TCM group, the Convention group (modern medicine treatment only), and the Integration group (including TCM combined with interventional or chemotherapy drugs), and the data of the three groups were simply compared. As shown in Fig. 3, the median OS of the Integration group was higher than that of the Convention group and the TCM group, which was 16.71 vs 15.11 vs 7.72 months (*P* > 0.05). We found that the combination of TCM and modern medicine in the treatment of HCC seemed to prolong the median OS of samples. However, because of the sparse data and wide variations in treatment duration and research design across clinical studies, our results have no statistical differences, which could not more objectively explain the advantages of integrated traditional Chinese and Modern medicine in the treatment of HCC. More sample sizes and studies will be needed in the future to verify this conclusion. For a list of the ingredients in each TCM compound prescription, see Table S6 in Additional File 1.

## Discussion

HCC is one of the intractable malignant tumors with rapid progression and short survival [1]. Most patients lose the chance of radical cure when diagnosed with HCC. The tumor in them cannot be removed and is prone to recurrence and metastasis, and only be treated with chemotherapy, targeted drugs and ICIs. Their median survival time is only 3 to 7 months [140]. The survival time of patients with advanced HCC can no longer be effectively improved. Furthermore, liver dysfunction and adverse reactions after treatment also limit the follow-up treatment of HCC [141]. Therefore, patients with HCC have many problems, such as short survival time, poor prognosis, the lack of mild and effective treatment and so on. Previous studies have found that the comprehensive treatment with TCM was effective for patients in the middle and advanced stages of HCC, which extended PFS to some extent, improved patients' quality of life and long-term OS by improving myelosuppression caused by chemotherapy, and gastrointestinal side effects, while it had a safety profile [142]. In the process of treating HCC with TCM, the anti-cancer ability of patients was improved [143]. Although TCM has a poorer role in killing hepatoma cells than chemotherapy and targeting drugs, the combination of TCM and modern medicine provides a direction for future clinical research.

**Fig. 4:**
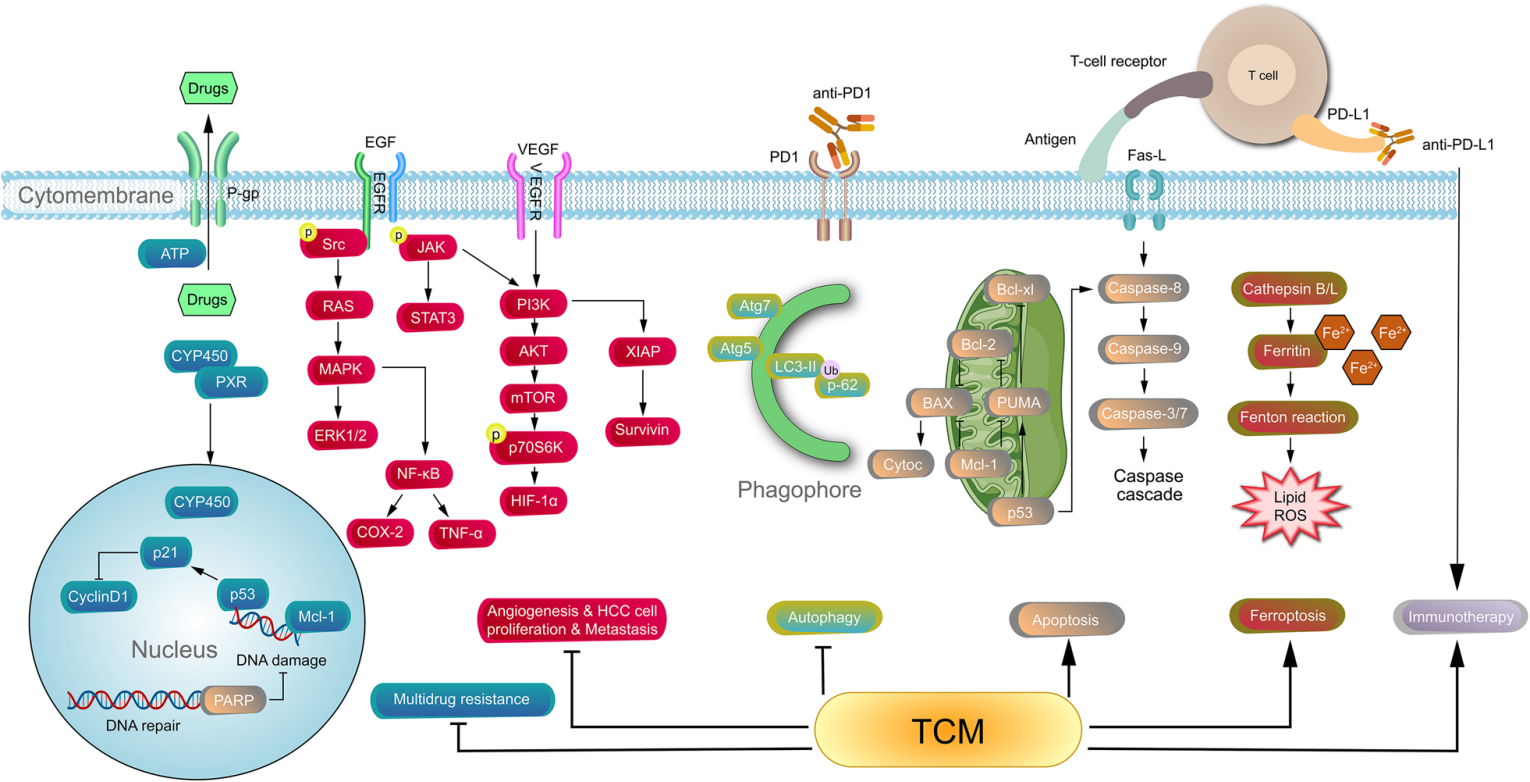
Related pathways and mechanisms involved in the treatment of HCC Integrated traditional Chinese and modern medicine inhibits MDR pathway by reversing P-gp. And through the EGFR and VEGF pathway inhibit the cell proliferation, tumor micro-vessel production and metastasis of HCC, regulate autophagy and induce apoptosis of hepatoma cells. TCM increases ROS production and induces ferroptosis in HCC cells. TCM promotes the curative effect of PD-1 or PD-L1, so as to enhance the immunity of tumor-bearing mice, improve the tumor micro-environment, realize the anti-tumor and improve drug resistance by enhancing chemotherapy, molecular targeted drugs and ICIs

This review systematically summarized the effect of TCM combined with clinical drugs and modern medicine therapy on the treatment of HCC. The basic researches included TCM combined with chemotherapy drugs, molecular targeted drugs and ICIs. This study found that integrated traditional Chinese and modern medicine inhibited MDR pathway by reversing P-gp. And through the EGFR and VEGF pathway inhibited the cell proliferation, tumor micro-vessel production and metastasis of HCC, regulated autophagy and induced apoptosis of hepatoma cells. TCM increased ROS production and induced ferroptosis in HCC cells. TCM promoted the curative effect of PD-1 or PD-L1, so as to enhance the immunity of tumor-bearing mice, improve the tumor micro-environment, realize the anti-tumor and improve drug resistance by enhancing chemotherapy, molecular targeted drugs and ICIs (Fig. 4).

In addition to summarizing the main effective mechanisms of TCM combined treatment for HCC, this review also evaluated the role of TCM in clinical researches. In HCC (early) surgery, TCM prolonged the median RFS of patients, reduced the recurrence rate after surgery, prolonged the OS [123–126]. In advanced HCC, TCM reduced gastrointestinal adverse reactions of other therapies, thereby improving the quality of life of patients [127–129]. TCM and IT had good synergistic effect, and improve the total effective rate, delay the progress of the disease [129, 131, 134]. But in this study, we found that integrated traditional Chinese and modern medical treatment of HCC did not form a systematic treatment system and the treatment principles were not really clear. Currently, symptomatic care makes up the majority of combined TCM and modern medicine treatment for HCC. If it can provide a relatively fixed, mild and effective TCM compound or proprietary Chinese medicine in each stage of HCC or in combination with various chemotherapy drugs, interventional therapy and immunotherapy, it will be more conducive to the treatment of clinicians and the survival of patients. Due to the HCC's complicated etiology, quick advancement, and patients' varied health statuses, there are frequently a lot of ambiguities in clinical therapy, which lead to a poor clinical therapeutic outcome when compares to the RCT research. Based on these problems, maybe real-world study could help understand the safety and effectiveness of integrated treatment of HCC, to obtain the evidence-based medicine evidence of TCM and provide a novel HCC treatment plan [144, 145].

This review introduced the molecular mechanism and clinical efficacy of TCM in the treatment of HCC and found that TCM played a positive role in the treatment of HCC in each stage and delayed the recurrence and metastasis of HCC, which laid a foundation for the further research and clinical use of HCC. But the deficiencies of the research were found. First off, this study did not examine the effectiveness or mechanism of action of TCM alone in the development of HCC; it only covered the combination therapy of TCM and contemporary medicine. Because TCM combined with modern medicine is a common choice for the treatment of advanced HCC. Secondly, only a small number of clinical studies (especially RCT) of TCM combined with modern medicine were reviewed, and most of them are retrospective studies, which might be related to the poor prognosis and short survival time of patients with HCC. Therefore, clinical researchers need to carry out multi-center clinical studies with a large sample size on patients with HCC and provide evidence-based medical evidence of TCM in the treatment of HCC. In addition, there were few studies on the combination of TCM and emerging therapy ICIs in the treatment of HCC. Previous studies have found that TCM improves the immune function of patients, which also points out a direction for further research.

## Conclusion

In summary, this review summarized experimental and clinical studies of TCM combined with modern medicine for the treatment of HCC, and TCM was found to synergize with modern medicine to inhibit the development of HCC, enhance sensitivity after chemotherapeutic resistance, improve adverse effects produced by chemotherapeutic agents and molecular targeted drugs, and prolong survival after surgery or interventional therapy. To some extent, it alleviates recurrence and metastasis of HCC and plays a role of attenuating cancer, which suggest that TCM treatment can serve as an adjuvant therapy to intervene the treatment of HCC. The paper provides a reference for the treatment and research of HCC in the future.

## Supplementary Information

**Additional file 1: Table S1.** Basic research on TCM combined with modern medical drugs in the treatment of HCC. **Table S2.** TCM overcome MDR. **Table S3. **TCM combine with Surgery for HCC. **Table S4. **TCM combined with IT for HCC. **Table S5.** TCM combined with CT for HCC. S6**. Table S6.** The components of TCM Formulas mentioned in this review.

## Data Availability

The data used for this study are included in the manuscript and additional file.

## Abbreviations

TCM: Traditional Chinese medicine
HCC: Hepatocellular carcinoma
IT: Interventional therapy
CT: Conventional therapy
ALD: Alcoholic liver disease
NAFLD: Non-alcoholic fatty liver disease
ICC: Intrahepatic cholangiocarcinoma
PS: Performance status
VEGF: Vascular endothelial growth factor
TLR4: Toll-like receptor 4
STAT3: Signal transducer and activator of transcription 3
PDGF: Platelet derived growth factor
RFA: Radiofrequency ablation
TACE: Transcatheter arterial chemoembolization
RCT: Randomized controlled trial
MMC: Mitomycin CDOX: doxorubicin
CTX: Cyclophosphamide
5-FU: 5-Fluorouracil
PES: Post embolism syndrome
ICIs: Immune checkpoint inhibitors
PD1: Anti-programmed cell death protein 1
PD-L1: Programmed cell death-Ligand 1
DHZCP: Dahuang Zhechong Pill
AE-SN: Aqueous extract of Solanum nigrum
HGK: Hydroxygenkwanin
NPPN: Neutral polysaccharide from Panax notoginseng
BSHYJD: Bushen huayu jiedu Recipe
WSTF: Water-soluble total flavonoids from Isodon lophanthoides var. gerardianus (Benth.) H. Hara
YIV-906: Huangqin Decoction
NF-κB: Nuclear Factor kappa B
Bcl-2: B-cell lymphoma-2
Bax: Bcl-2-associated X
Bcl-xl: B-cell lymphoma-extra large
TNF-α: Tumor necrosis factor-α
IL-6: Interleukin-6
IL-2: Interleukin-2
OS: Overall survival
Fas: Factor associated suicide
FasL: Factor associated suicide ligand
LC-3: Light chain-3
PI3K: Phosphatidylinositol 3 kinase
mTOR: Mammalian target of rapamycin
PLD: Pegylated liposomal DOX
ERK: Extracellular regulated protein kinases
CRT: CalreticulinHMGB1: high mobility group box 1
MMP: Matrix metalloproteinase
PTTG1: Pituitary tumor-transforming 1
MRP2: Multidrug resistance-associated protein 2
CYR61: Cysteine-rich61
OX1R: Orexin 1 receptor
GHSR: Growth hormone secretagogue receptor
NPY: Neuropeptide Y
AGRP: Agouti related neuropeptide
Ob-R: Leptin receptor
POMC: Pro-opiomelanocortin
CART: Cocaine-and amphetamine-regulated transcript
P-gp: P-glycoprotein
CM: Condition medium
CCRK: Cell cycle related kinase
TCF: Tcell factor
TNFR: Tumor necrosis factor receptor
MAPK: Mitogen-activated protein kinase
JAK: Janus Kinase
E-Cad: E-cadherin
Vim: Vimentin
C-FLIP: Cellular FLICE-like inhibitory protein
ECM: Extracellular matrix
MKK4: Mitogen-Activated Protein Kinase Kinase 4
JNK: C-Jun N-terminal kinase
uPA: Urokinase plasminogen activator
HepG2-SR: Sorafenib-resistant HCC cell line
LDH: Lactate dehydrogenase
HIF-1α: Hypoxic inducible factor 1α
FASN: Fatty Acid Synthase
HDL-C: High density liptein cholesterol
EMT: Epithelial–mesenchymal transition
IL1: Interleukin-1
MyD88: Myeloiddifferentiationfactor88
IκB-α: Inhibitor of NF-κB alpha
EGF: Epithelial growth factorHCPT: 10-Hydroxycamptothecin
MCP-1: Monocyte chemoattractant protein-1
CXCL: Chemokine (C-X-C motif) Ligand
TLR7: Toll-like receptor 7
VEGFR: Vascular endothelial growth factor receptor
R-HepG2: Multidrug resistant HCC cell line
AANG: Asiatic acid Naringenin
TGF-β: Transforming growth factor-β
Smad: Intracellular effectors of TGF-β signalling
HSP90: Heat shock protein 90
HDACi: Histone deacetylase inhibitor
HDACi-R: HDACi-resistant
THM: Traditional Herbal Medicine
ALT: Alanine transaminase
AST: Aspartate transaminase
DFS: Disease-free survival
JLD: Jian Pi Li gan decoction
CHHJ: Chaihu-Huaji decoction
FFBM: Compound cantharidin capsules
JLC: Jinlong capsule
JD: Jie-du granules
SOR: Sorafenib
CHM: Chinese herbal medicine
HR: Hazard ratio
95%CI: 95% Confidence interval
